# Boredom proneness and short-video addiction among Chinese vocational college students: psychological resilience as a mediator and gender as a moderator

**DOI:** 10.3389/fpsyg.2026.1889736

**Published:** 2026-07-31

**Authors:** Xueli Hui, Yan Wang, Wenfeng Cheng, Yantao Shi

**Affiliations:** 1 Faculty of Education, Guangxi Normal University, Guilin, China; 2 College of Preschool Education, Chongqing Youth Vocational & Technical College, Chongqing, China; 3 College of Ethnology and Sociology of Guangxi Minzu University, Nanning, China; 4 College of Innovation and Entrepreneurship, Guangxi Minzu University, Nanning, China

**Keywords:** boredom proneness, gender, psychological resilience, short-video addiction, vocational college students

## Abstract

**Objective:**

This study examined the mechanism linking boredom proneness to short-video addiction among vocational college students, with psychological resilience as a mediator and gender as a moderator.

**Design:**

A cross-sectional survey design was used to examine whether psychological resilience mediated the association between boredom proneness and short-video addiction, and whether gender moderated the direct and indirect pathways.

**Methods:**

A questionnaire survey was conducted among 2,687 vocational college students in Guangxi, China, aged [18–21] years (M = 19.28, SD = 1.21). Measures of boredom proneness, psychological resilience, and short-video addiction were collected. Data were analyzed using PROCESS Model 58.

**Results:**

Boredom proneness positively predicted short-video addiction. Psychological resilience partially mediated this relationship, indicating that boredom proneness not only directly increased short-video addiction but also indirectly increased it by diminishing psychological resilience. Gender significantly moderated this indirect pathway. Compared with female students, boredom proneness showed a stronger negative association with psychological resilience among male students. In addition, the protective effect of psychological resilience on short-video addiction was significant only among males, but not among females. The index of moderated mediation was significant, confirming gender differences in the indirect effect.

**Conclusion:**

Boredom proneness influences short-video addiction through both direct and indirect pathways, and this mechanism varies by gender. These findings deepen understanding of how boredom proneness contributes to short-video addiction among vocational college students.

## 1 Introduction

Short-video platforms have become deeply embedded in young people's everyday lives, partly due to their distinctive media affordances, including short-form content, high sensory stimulation, immediate feedback, and algorithmically personalized recommendations (Zhang N. et al., 2023; Ye et al., 2022). Short-video addiction refers to a problematic pattern of excessive and poorly controlled use of short-video content, characterized by persistent urges to use, impaired self-regulation, and disruptions to learning, daily functioning, and psychological wellbeing (Qin et al., 2019; Zhang N. et al., 2023; Ye et al., 2023). Accumulating empirical and review evidence indicates that problematic short-video use is associated with a range of maladaptive outcomes, including reduced learning motivation and wellbeing among vocational college students, lower learning engagement, perceived learning inefficacy, academic procrastination partly related to impaired attentional control, and broader mental-health difficulties associated with problematic TikTok or short-video use (Ye et al., 2022, 2023; Xie et al., 2023; Jain et al., 2025).

Short videos are particularly prevalent among college students, who often use them to satisfy needs related to academic learning, self-expression, entertainment, and social connection, while also being at risk of excessive or dysregulated use (Jain et al., 2025; Zhang N. et al., 2023). The present study focuses on vocational college students, a group that may be especially susceptible to problematic short-video use because of lower perceived academic demands in some contexts, greater discretionary time, weaker learning motivation, heightened classroom boredom, and limited self-regulatory resources. Compared with general university students, vocational college students are embedded in a more practice-oriented and employment-driven educational context, where learning engagement, practical training, and perceived learning effectiveness are closely related to students' academic adaptation and wellbeing (Ye et al., 2022, 2023). Repetitive skill training, career-readiness pressure, and relatively unstable academic engagement may be associated with greater vulnerability to boredom and a stronger tendency to use short videos for stimulation and emotion regulation, consistent with the compensatory internet use framework (Camerini et al., 2023; Kardefelt-Winther, 2014). Such problematic use may be associated with poorer attention, weaker self-control, and lower learning engagement, thereby potentially affecting skill acquisition, practical training quality, and future career development (Li et al., 2024; Xie et al., 2023). Therefore, examining vocational college students extends existing research to a practice-oriented vocational education context and provides more targeted evidence for understanding and addressing boredom-related short-video addiction. Nevertheless, existing research has primarily examined general college students and young adults, with insufficient attention to the psychological antecedents and underlying mechanisms of short-video addiction among vocational college students. Targeted empirical investigation of this population is therefore warranted.

Among the numerous psychological factors related to short-video addiction, boredom proneness may be an important emotional susceptibility trait. Boredom is generally defined as an unpleasant low-arousal emotional state characterized by insufficient stimulation, difficulty concentrating, and a reduced sense of meaning in the current activity (Danckert and Allman, 2005). Boredom proneness refers to a relatively stable characteristic that reflects an individual's tendency to experience boredom with greater frequency and intensity, typically manifested as low tolerance for repetitive tasks, impaired attentional control, reduced intrinsic motivation, and a diminished sense of purpose (Eastwood et al., 2012; Malkovsky et al., 2012; Westgate and Wilson, 2018). From a cognitive perspective, boredom proneness may reduce students' attention to current tasks and increase their preference for activities that provide immediate stimulation and rapid feedback (Tagliaferri et al., 2025; Tam and Inzlicht, 2024). This trait is more likely to emerge in repetitive, monotonous, and novelty-poor task environments (Pawlak et al., 2020; Kruk et al., 2021; Perkins and Hill, 1985). For vocational college students, the repetitiveness of study tasks, the continuity of practical training, and career development pressure may be associated with heightened boredom, which may further be related to a greater propensity to engage with short-video content (Xie et al., 2023; Li et al., 2024). Previous studies have shown that boredom proneness is significantly positively correlated with problematic use of digital media (Camerini et al., 2023; Elhai et al., 2018; Wolniewicz et al., 2020; Fioravanti et al., 2024; Zhou et al., 2025). According to the compensatory internet use framework, negative experiences such as boredom and stress may lead individuals to engage in online activities as a form of emotion regulation and psychological compensation, thereby being linked to greater susceptibility to problematic internet behaviors (Kardefelt-Winther, 2014). Consistent with this theoretical proposition, meta-analytic evidence indicates a significant positive association between boredom proneness and maladaptive digital media use (Camerini et al., 2023). Therefore, boredom proneness may not only serve as an emotional backdrop for frequent short-video use, but may also be strongly associated with problematic short-video use.

In conclusion, existing studies have laid a foundation for understanding the association between boredom proneness and short-video addiction, but several limitations remain: Firstly, vocational college students constitute an underrepresented population in the scholarly literature, despite facing substantial pressures related to academic adaptation, skill acquisition, and career readiness. Compared with general university students, their practice-oriented and career-focused learning context offers a more specific perspective for understanding boredom-related problematic short-video use. Second, limited empirical attention has been paid to the protective psychological resources that may buffer or explain the association between boredom proneness and short-video addiction. Third, relatively little research has examined whether this psychological process is moderated by individual differences, such as gender. To address these gaps, the present study uses boredom proneness as the independent variable and psychological resilience as the mediating variable to construct a moderated mediation model. Specifically, it examines whether psychological resilience mediates the association between boredom proneness and short-video addiction and whether gender moderates this process. This study extends short-video addiction research to vocational college students, clarifies the protective role of psychological resilience, and offers practical implications for targeted digital-behavior interventions in vocational education.

### 1.1 Boredom proneness and short-video addiction tendency

Boredom proneness may be closely associated with short-video addiction. According to the I-PACE model, addictive application use can be understood as the result of interactions between individual susceptibility factors and situation-specific emotional and cognitive processes. This process may be associated with weakened self-control and reinforcement learning, thereby contributing to the maintenance of problematic use (Brand et al., 2019; Tie et al., 2025; Zhan et al., 2025). In this process, students with high boredom proneness may cognitively evaluate short videos as a convenient way to escape monotony, restore arousal, and obtain immediate rewards (Tagliaferri et al., 2025; Xie et al., 2023).

Boredom proneness is one of the individual susceptibility factors and is related to more frequent experiences of low-stimulation emotional states and stronger motivation to seek immediate rewards and emotional relief (Cannito et al., 2023; Tagliaferri et al., 2025; Tam and Inzlicht, 2024; Wan et al., 2025). Relevant studies have shown that individuals with higher boredom proneness are more likely to experience boredom across different situations, with core characteristics including insufficient stimulation and inadequate attention investment (Danckert and Elpidorou, 2023; Gorelik and Eastwood, 2024; Ng et al., 2024). Further studies have shown that individuals with higher boredom proneness may be more sensitive to “quick, low-cost, and immediately available” rewards in low-arousal negative states (Tagliaferri et al., 2025). Short-video platforms provide immediate rewards through frequent updates and algorithmic recommendations, which may quickly enhance arousal and provide temporary relief from boredom, thereby being associated with individuals' tendency to use short videos for emotion regulation (Cheng et al., 2023; Liao, 2024). Furthermore, existing studies indicate that boredom proneness is related to individuals' use of short videos to mitigate boredom and may be associated with weakened self-control, habitual use, and addictive behaviors such as loss of control and functional impairment (Zhang et al., 2024). Based on the above theoretical and empirical evidence, this study proposes the following hypothesis:

H1: Boredom proneness is significantly positively correlated with short-video addiction.

### 1.2 Mediating role of psychological resilience

Psychological resilience is typically defined as a process and capacity that enables individuals to adapt flexibly, cope effectively, and recover from stress, adversity, or disruptive life events (Luthar et al., 2000; Bonanno et al., 2024). In the present study, psychological resilience is conceptualized as a distinct recovery-oriented personal resource rather than as a proxy for self-control, stress symptoms, or psychopathology. Empirical work has suggested that boredom proneness is associated with poorer psychological adjustment, diminished wellbeing, and lower resilience-related functioning (Lee and Zelman, 2019; Tutzer et al., 2021; Akyil, 2025). From a Conservation of Resources perspective, individuals strive to acquire, maintain, and protect valued resources, and psychological strain occurs when these resources are threatened, depleted, or insufficiently replenished (Hobfoll, 2015; Hobfoll et al., 2018). This theory does not require boredom and resilience to be treated as identical constructs; rather, it provides a resource-based framework for explaining why repeated exposure to low-stimulation and low-meaning experiences may be associated with weaker adaptive resources. Students with higher boredom proneness may repeatedly experience attentional disengagement, low perceived meaning, and reduced goal involvement, which may be associated with a reduced capacity to recover from stress and maintain adaptive functioning. Direct empirical evidence also supports this inference: Akyil (2025) reported a significant negative association between boredom proneness and resilience, indicating that individuals who are more prone to boredom tend to report lower resilience.

Based on the above theoretical and empirical evidence, this study hypothesizes the following:

H2a: There is a significant negative correlation between boredom proneness and psychological resilience.

Psychological resilience is significantly negatively associated with students' short-video addiction. According to the Conservation of Resources (COR) theory, the sufficiency of psychological resources is related to individuals' abilities in stress coping, emotion regulation, and behavioral control. Psychological resilience, as a crucial protective resource, may help students maintain positive adaptation when facing academic pressure and boredom, thereby reducing their tendency to seek immediate compensation or emotional escape through short videos (Hou et al., 2017; Ji et al., 2025). As a protective mechanism, psychological resilience may help students regulate boredom more adaptively, maintain behavioral control, and reduce their reliance on short videos for immediate emotional relief (Wu et al., 2024). Relevant studies have shown that individuals with higher psychological resilience may be less likely to rely on media with immediate reward attributes, such as short videos, for emotional compensation (Hou et al., 2017). Compared with individuals with high psychological resilience, those with low resilience may be more likely to rely on the immediate reward mechanisms of short-video platforms because of their relatively limited capacity for psychological resource recovery, and may exhibit elevated levels of short-video addiction (Xue et al., 2025; Brand et al., 2019). Empirical studies have demonstrated a negative association between psychological resilience and short-video addiction, and research indicates that psychological resilience is a significant negative predictor of individuals' addictive media-use behaviors (Xue et al., 2025; Hou et al., 2017; Ji et al., 2025). Furthermore, studies focusing on student populations have also reported a significant negative correlation between psychological resilience and short-video addiction. In other words, higher levels of psychological resilience are associated with a lower propensity for students to engage in short-video addictive behaviors (Robertson et al., 2018). Accordingly, this study proposes the following hypothesis:

H2b: There is a significant negative correlation between psychological resilience and students' short-video addiction.

Based on the above research, psychological resilience may serve as a statistical mediator between boredom proneness and short-video addiction. Boredom proneness is usually associated with lower levels of self-regulation, adaptive coping abilities, and psychological resources, and may be related to lower psychological resilience. Meanwhile, psychological resilience, as an internal protective resource, may be associated with individuals' ability to maintain adaptive functioning when facing boredom, stress, or negative emotions, and may be linked to a lower tendency to compensate for these experiences through online media use. Previous studies have shown that psychological resilience is significantly negatively correlated with problematic internet use behavior among adolescents (Zhou et al., 2017; Robertson et al., 2018), indicating that psychological resilience may serve as an important protective psychological resource connecting negative psychological traits and addictive media use. Although direct evidence examining this mediating pathway remains limited, existing evidence has provided indirect support for this inference. Based on this, this study proposes the following hypothesis:

H2: Psychological resilience mediates the association between boredom proneness and short-video addiction.

### 1.3 Moderating role of gender

First, existing research has shown that boredom proneness is significantly associated with psychological resilience (Akyil, 2025; Lee and Zelman, 2019; Yan et al., 2021; Bieleke et al., 2022). However, relatively few studies have examined whether this relationship varies by gender, especially among vocational college students. Gender-role socialization theory proposes that, throughout development, men and women may be exposed to distinct social expectations, normative roles, and behavioral demands, leading them to gradually develop differentiated patterns of emotional expression, stress coping, and support seeking (Rosario et al., 1988; Sigmon et al., 1995; Ayllón-Negrillo et al., 2024). Therefore, gender may be associated with individuals' perceptions of boredom and their coping strategies. This perspective supports the first-stage moderation because gendered differences in emotional expression, support seeking, and coping with boredom may alter the association between boredom proneness and psychological resilience. Specifically, women tend to cope with negative experiences through emotional expression, interpersonal communication, and support seeking (Tamres et al., 2002; Nolen-Hoeksema and Aldao, 2011; Burleson, 2003; Tifferet, 2020), whereas men are more likely to adopt coping strategies such as emotional suppression, problem avoidance, or relative detachment when facing stress (Matud, 2004; Flynn et al., 2010). Because boredom proneness is typically associated with low stimulation, impaired attentional control, and a diminished sense of activity significance, these experiences may be related to lower self-regulatory capacity and fewer psychological adaptation resources (Lee and Zelman, 2019; Akyil, 2025). Therefore, compared with female students, male students may experience greater difficulty offsetting boredom-related psychological strain through emotional expression and social support, which may strengthen the negative association between boredom proneness and psychological resilience. In other words, first-stage moderation is theoretically justified because gendered coping patterns may shape how strongly boredom proneness is associated with lower psychological resilience. Based on the above theoretical derivation, this study proposes the following hypothesis:

H3a: Gender moderates the relationship between boredom proneness and psychological resilience. Specifically, compared to female vocational college students, the negative association between boredom proneness and psychological resilience is expected to be stronger among male vocational college students.

Second, the Protective Factor Model offers a useful basis for explaining the role of psychological resilience in short-video addiction. This model suggests that maladaptive outcomes may be associated with the interplay between risk exposure and protective resources (Efrati et al., 2023; Gubbels et al., 2024). In this study, gender is not treated as either a risk factor or a protective factor. Instead, it is examined as a demographic boundary condition that may influence the strength of the association between psychological resilience and short-video addiction. This supports the second-stage moderation because resilience may be more strongly linked to short-video addiction when students rely primarily on internal self-regulation rather than external emotional or interpersonal support.

Psychological resilience may be associated with students' ability to regulate negative emotions, maintain behavioral control, and recover from stressful or boring experiences. Therefore, students with higher resilience may be less likely to rely on short videos for immediate emotional relief or compensation (Hou et al., 2017; Hao et al., 2022). This interpretation is consistent with previous evidence showing that resilience is negatively associated with problematic internet use and problematic smartphone use (Hidalgo-Fuentes et al., 2023; Yam et al., 2024).

The association between resilience and short-video addiction may also differ by gender. Gender-role socialization is closely related to individuals' emotional expression, coping strategies, and support-seeking behaviors (Matud, 2004; Tamres et al., 2002). Prior research suggests that women are generally more likely to use emotional expression and interpersonal support, whereas men are more likely to rely on restrained emotional expression or internal coping strategies (Nolen-Hoeksema and Aldao, 2011; Tifferet, 2020). Among vocational college students, short-video use may serve several functions, including entertainment, emotion regulation, social connection, and stress relief. Accordingly, the association between psychological resilience and short-video addiction may differ by gender because male and female students may use short videos for different psychological and social purposes.

Male students who experience boredom, stress, or negative emotions may be less likely to seek interpersonal support or use expressive coping strategies. As a result, they may depend more on internal regulatory resources, such as psychological resilience, to regulate their media use. In this context, psychological resilience may show a stronger negative association with short-video addiction among male students. By contrast, female students' short-video use may be more closely linked to interpersonal connection, emotional expression, and social interaction, which may reduce the independent negative association between resilience and short-video addiction. Thus, the second-stage moderation is theoretically expected because gender may influence the extent to which psychological resilience is independently linked to problematic short-video use. Therefore, the negative association between psychological resilience and short-video addiction is expected to be stronger among male students than among female students. Based on this reasoning, the following hypothesis is proposed:

H3b: Gender moderates the relationship between psychological resilience and short-video addiction. Specifically, the negative association between psychological resilience and short-video addiction is expected to be stronger among male vocational college students than among female vocational college students.

### 1.4 The purpose of this study

In summary, this study examines the relationship between boredom proneness and short-video addiction among vocational college students and tests the mediating role of psychological resilience and the moderating effect of gender. Specifically, this study examines whether boredom proneness is associated with lower psychological resilience and higher short-video addiction, and whether this indirect association varies by gender. By constructing a moderated mediation model, this study explores the potential psychological processes underlying short-video addiction among vocational college students from the perspective of “risk experience–protective psychological resources–problematic media use.” Given the cross-sectional design of this study, the findings should be interpreted as evidence of associations rather than causal mechanisms. The results may deepen the understanding of factors associated with problematic short-video use among vocational college students and provide empirical evidence for strengthening psychological resilience, improving emotion regulation abilities, and promoting healthy media use education.

## 2 Methods

### 2.1 Participants

This study specifically targeted students actively enrolled in vocational college in China, establishing this as our primary inclusion criterion. The exclusion criterion was set as involuntary participation in this study. Using a convenient sample, students were invited to complete a questionnaire through the Questionnaire Star (www.wjx.cn). A total of 3,000 students were initially enrolled to participate in the survey. After excluding invalid submissions, a total of 2,687 valid questionnaires were collected, yielding an effective response rate of 89.56%. The sample included 1,986 females (73.9%) and males 701 (26.1%). The average age was 19.28 (SD = 1.21), ranging from 18 to 21. With regard to their geographical origin, 347 (12.9%) were from cities, a total of 2,340 (87.1%) originate from rural regions. With respect to their grade level in academia, 37.4% are currently in their first year of vocational college, 47.3% are currently in their second year of vocational college, and 15.2% are currently in their third year of vocational college. The potential influence of the uneven sample distribution, particularly in terms of gender and geographical origin, is further discussed in the Limitations section.

### 2.2 Procedure

This study was approved by the Ethics Committee of the authors' university. Before data collection, all participants provided written informed consent and were informed of the study purpose, voluntary participation, anonymity, confidentiality, and their right to withdraw at any time. Data were collected through a widely used online survey platform in China (www.wjx.cn). Participants first completed demographic questions, including gender, age, and year of study, and then completed the measures of boredom proneness, psychological resilience, and short-video addiction. All questionnaires were presented in the same order, and the average completion time was approximately 5–10 min. The research team had no direct contact with participants during questionnaire completion, which helped reduce potential interviewer influence and protect participants' privacy.

After data collection, the data were screened for invalid responses, including incomplete questionnaires, patterned responses, and unrealistically short completion times. Descriptive statistics, reliability analyses, and confirmatory factor analyses were then conducted to examine the basic characteristics and psychometric properties of the measures. Pearson correlation analyses were used to examine bivariate associations among the study variables. Finally, mediation and moderated mediation analyses were conducted using the PROCESS macro for SPSS, with age and year of study included as control variables. The significance of indirect and conditional indirect effects was tested using 5,000 bootstrap resamples.

### 2.3 Measures

#### 2.3.1 Boredom proneness

Boredom proneness was assessed using the 12-item Boredom Proneness Scale–Short Form (BPS-SF; Vodanovich et al., 2005). Although previous research has validated a related short boredom proneness measure in Chinese college students (Peng et al., 2020), the present study further examined the reliability and construct validity of the 12-item BPS-SF in vocational college students. The scale consists of two dimensions: internal stimulation and external stimulation, with six items for each dimension. Sample items include “I find it easy to entertain myself” and “It would be very hard for me to find a job that is exciting enough.” Each item was rated on a seven-point Likert scale, with higher mean scores indicating higher levels of boredom proneness after reverse-coded items were recoded. The second-order CFA model showed acceptable construct validity, CMIN = 462.185, df = 43, χ^2^/df = 10.748, GFI = 0.974, NFI = 0.972, TLI = 0.961, CFI = 0.975, RMSEA = 0.060. Although χ^2^/df was relatively high, this index is sensitive to large sample size, whereas the other fit indices indicated acceptable model fit. In this study, the overall scale showed satisfactory internal consistency, Cronbach's α = 0.844.

#### 2.3.2 Short-video addiction

The College Students' Short Video Addiction Scale (CS-SVAS; Qin et al., 2019) was used to assess short-video addiction among vocational college students. This scale was originally developed for Chinese college students and was adapted from established measures of mobile-phone and Internet addiction. The scale includes 14 items across four dimensions: withdrawal (five items; e.g., “If you don't watch short videos, you will feel restless”), avoidance or escapism (three items; e.g., “When you're feeling down, you watch short videos to lift your mood”), loss of control (four items; e.g., “You find yourself spending more time on short videos than you intended”), and inefficiency (two items; e.g., “You find yourself indulging in short videos when you should be doing other necessary tasks, which brings you some trouble”). Each item was rated on a five-point Likert scale ranging from 1 (completely inconsistent) to 5 (completely consistent), with higher mean scores indicating higher levels of short-video addiction. The second-order CFA model showed that the SAC had good construct validity, with CMIN = 455.405, DF = 65, χ^2^/df = 7.006, GFI = 0.976, NFI = 0.977, TLI = 0.973, CFI = 0.980, and RMSEA = 0.047. In this study, the overall scale's internal consistency reliability coefficient is 0.888.

#### 2.3.3 Psychological resilience

Psychological resilience was measured using the six-item Brief Resilience Scale (BRS; Smith et al., 2008), which has been adapted and validated among Chinese undergraduate samples from Hong Kong and mainland China and has shown acceptable reliability and validity (Lai and Yue, 2014). The BRS assesses individuals' ability to recover from stress and adversity and includes positively worded items, such as “It does not take me long to recover from a stressful event,” and negatively worded items, such as “It is hard for me to snap back when something bad happens.” Each item was rated on a five-point Likert scale ranging from 1 = strongly disagree to 5 = strongly agree, with higher mean scores indicating higher psychological resilience after reverse-coded items were recoded. In the present study, the scale showed acceptable construct validity, CMIN = 50.476, df = 7, χ^2^/df = 7.211, GFI = 0.994, NFI = 0.989, TLI = 0.980, CFI = 0.991, RMSEA = 0.048, and acceptable internal consistency, Cronbach's α = 0.724.

### 2.4 Data analysis

First, descriptive statistics were calculated for the main study variables, and Pearson correlation analyses were conducted to examine the associations among boredom proneness, psychological resilience, short-video addiction, and gender (see Table 1). Before testing the mediation and moderated mediation models, diagnostic tests were performed to examine the statistical assumptions underlying regression-based analyses. Specifically, linearity and homoscedasticity were assessed using scatterplots of standardized residuals against standardized predicted values. The distributional characteristics of the main continuous variables were evaluated using skewness and kurtosis. Multicollinearity was examined using tolerance values and variance inflation factors (VIFs). The results showed that skewness values for the main continuous variables ranged from −0.498 to 0.810, and kurtosis values ranged from 0.119 to 1.942, both within acceptable ranges. All VIF values were below 1.77, and all tolerance values were above 0.565, indicating no serious multicollinearity. In addition, the standardized residual plots showed no obvious curvilinear pattern or funnel-shaped distribution, suggesting that the assumptions of linearity and homoscedasticity were generally satisfied.

**Table 1:** Correlations among study variables.

Variables	M	SD	1	2	3	4	5	6
1. Gender	—	—	1					
2. Year of study	—	—	−0.066^**^	1				
3. Age	19.280	1.206	−0.079^**^	0.585^**^	1			
4. Boredom proneness	4.619	0.817	−0.141^**^	0.048^*^	0.039^*^	1		
5. Short-video addiction	3.346	0.714	−0.180^**^	0.080^**^	0.053^**^	0.195^**^	1	
6. Psychological resilience	2.550	0.546	0.092^**^	−0.021	−0.050^**^	−0.485^**^	−0.137^**^	1

^*^p < 0.05, ^**^p < 0.01.

**Figure 1:**
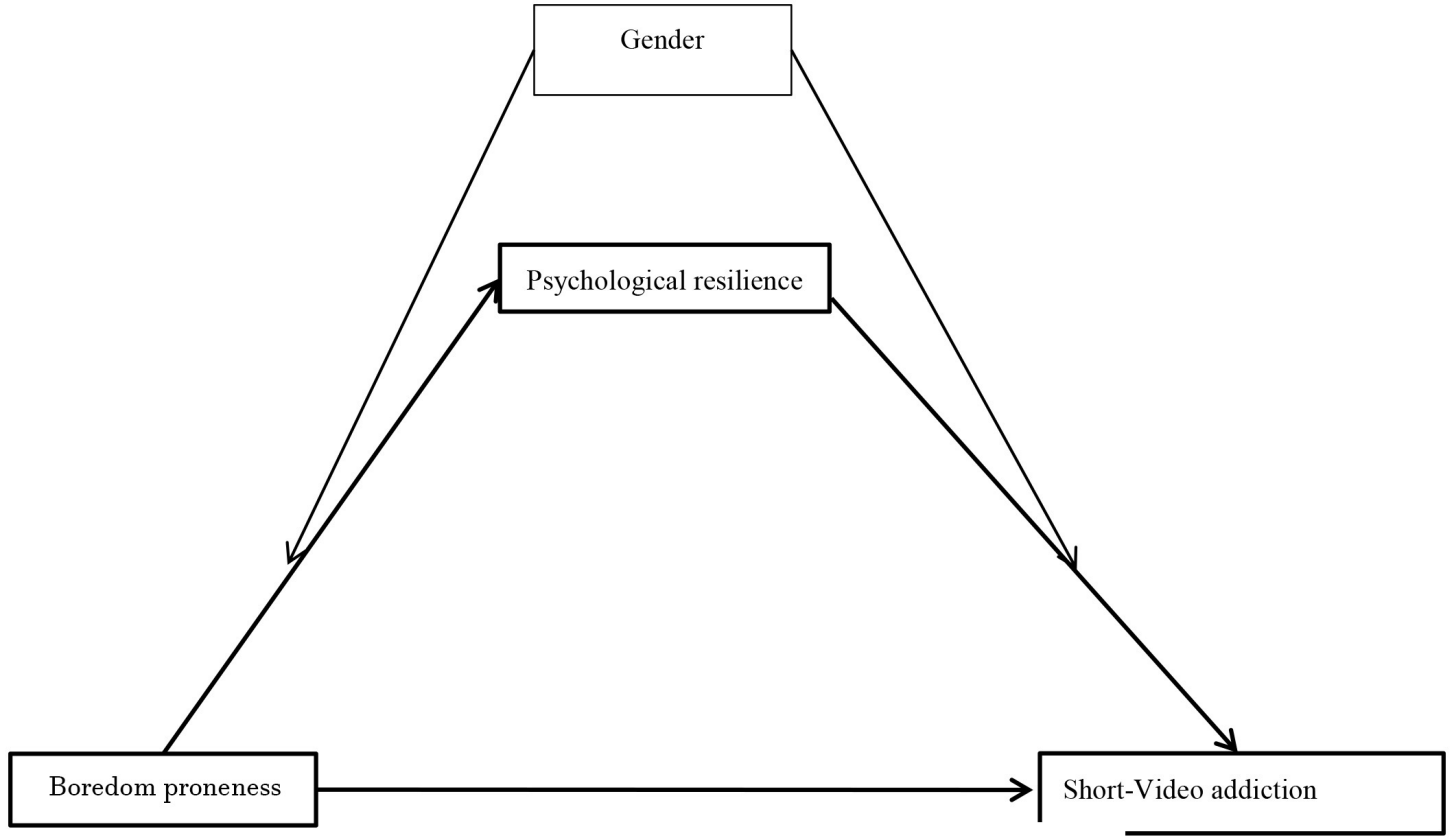
The assumed moderated mediation model.

Second, the mediating role of psychological resilience in the association between boredom proneness and short-video addiction was tested using Model 4 of the PROCESS macro developed by Hayes (2013; see Table 2; Figure 1). Model 58 of the PROCESS macro was then used to examine whether gender moderated the path from boredom proneness to psychological resilience and the path from psychological resilience to short-video addiction, thereby testing whether the conditional indirect association between boredom proneness and short-video addiction through psychological resilience differed by gender (see Table 3; Figure 1). Bootstrapping procedures with 5,000 resamples were used to test the significance of the direct, indirect, and conditional indirect associations, and 95% bias-corrected confidence intervals were reported. Associations were considered statistically significant when the confidence intervals did not include zero.

**Table 2:** Baseline indirect-association analysis.

Variables	Model 1 (Psychological resilience)				Model 2 (Short-video addiction)				Model 3 (Short-video addiction)			
	β	SE	LLCI	ULCI	β	SE	LLCI	ULCI	β	SE	LLCI	ULCI
Year of study	0.046	0.030	−0.012	0.105	0.101^**^	0.033	0.034	0.168	0.098^**^	0.033	0.031	0.164
Age	−0.041^*^	0.017	−0.075	−0.007	0.002	0.019	−0.035	0.040	0.004	0.019	−0.033	0.042
Boredom proneness	−0.485^***^	0.016	−0.518	−0.452	0.164^***^	0.022	0.122	0.206	0.195^***^	0.019	0.158	0.229
Psychological resilience	—	—	—	—	−0.056^**^	0.021	−0.098	−0.013	—	—	—	—
*R* ^2^	0.236				0.045				0.043			
*F*	278.22^**^				31.92^***^				40.25^***^			

β are standardized coefficients. SE, standard error; LLCI, lower limit of the 95% confidence interval; ULCI, upper limit of the 95% confidence interval.

^*^p < 0.05, ^**^p < 0.01, ^***^p < 0.001.

**Table 3:** Testing the moderated mediation effect of boredom proneness on Short-Video addiction.

Variables	Model 1 (Psychological resilience)				Model 2 (Short-video addiction)			
	β	SE	LLCI	ULCI	β	SE	LLCI	ULCI
Year of study	0.045	0.030	−0.013	0.104	0.091^**^	0.033	0.025	0.156
Age	−0.040^*^	0.017	−0.074	−0.006	−0.005	0.019	−0.042	0.033
Boredom proneness	−0.651^***^	0.060	−0.769	−0.533	0.138^***^	0.022	0.096	0.180
Gender	0.039	0.039	−0.037	0.116	−0.330^***^	0.043	−0.414	−0.245
Boredom proneness × Gender	0.103^**^	0.035	0.034	0.173	—	—	—	—
Psychological resilience	—	—	—	—	−0.339^***^	0.069	−0.476	−0.203
Psychological resilience	—	—	—	—	0.171^***^	0.039	0.094	0.249
*R* ^2^	0.239				0.070			
*F*	169.54^***^				35.68^***^			

β are standardized coefficients. SE, standard error; LLCI, lower limit of the 95% confidence interval; ULCI, upper limit of the 95% confidence interval. Each column is a regression model that predicts the criterion at the top of the column.^*^p < 0.05, ^**^p < 0.01, ^***^p < 0.001.

The PROCESS macro was selected because this study focused on testing mediation and moderated mediation using observed variables, rather than estimating a full latent-variable measurement model. Compared with structural equation modeling, the PROCESS macro provides a more direct and transparent approach to estimating specific indirect and conditional indirect associations and evaluating their significance using bootstrap confidence intervals. Therefore, it was considered appropriate for the hypothesis-driven analytical model used in this study.

## 3 Results

### 3.1 Common method bias test

Because the data were obtained via self-reported measures, the possibility of common method bias was examined using Harman's one-factor test. The analysis revealed 7 factors with eigenvalues greater than 1, and the first factor accounted for 25.30% of the variance, which is well below the 40% threshold. This indicates that common method bias was not significant.

### 3.2 Bivariate correlation analysis

Results of the bivariate correlation analyses for the variables of interest are presented in Table 1. Boredom proneness was positively associated with short-video addiction (*r* = 0.195, *p* < 0.01), and negatively associated with psychological resilience (*r* = −0.485, *p* < 0.01). Boredom proneness was also weakly and negatively correlated with gender (*r* = −0.141, *p* < 0.01) and weakly and positively correlated with age (*r* = 0.039, *p* < 0.05). Psychological resilience was negatively correlated with short-video addiction (*r* = −0.137, *p* < 0.01) and age (*r* = −0.050, *p* < 0.01), but positively correlated with gender (*r* = 0.092, *p* < 0.01). Additionally, gender showed a weak negative correlation with short-video addiction (*r* = −0.180, *p* < 0.01), whereas age showed a weak positive correlation with short-video addiction (*r* = 0.053, *p* < 0.01). Year of study was also significantly correlated with boredom proneness and short-video addiction. Therefore, age and year of study were included as control variables in the subsequent model analyses to reduce the potential influence of developmental stage and educational experience on the associations among boredom proneness, psychological resilience, and short-video addiction. Accordingly, Hypothesis 1 was supported.

### 3.3 Psychological resilience as a mediating mechanism

We used Model 4 of the PROCESS macro (Hayes, 2013) to examine whether psychological resilience mediated the association between boredom proneness and short-video addiction among vocational college students, with year of study and age included as control variables (see Figure 1). As shown in Table 2, after controlling for year of study and age, boredom proneness was negatively associated with psychological resilience (β = −0.485, SE = 0.016, 95% CI [−0.518, −0.452], *p* < 0.001; Model 1). Psychological resilience, in turn, was negatively associated with short-video addiction (β = −0.056, SE = 0.021, 95% CI [−0.098, −0.013], *p* < 0.01; Model 2). In addition, boredom proneness was positively associated with short-video addiction before the mediator was entered into the model (β = 0.195, SE = 0.019, 95% CI [0.158, 0.229], *p* < 0.001; Model 3). After psychological resilience was included, the direct association between boredom proneness and short-video addiction remained significant (β = 0.164, SE = 0.022, 95% CI [0.122, 0.206], *p* < 0.001; Model 2).

Using 5,000 bootstrap samples, the indirect association between boredom proneness and short-video addiction through psychological resilience was significant (indirect effect = 0.027, SE = 0.013, 95% CI [0.001, 0.053]). Given that the direct association between boredom proneness and short-video addiction remained significant after the inclusion of psychological resilience, the results indicate a partial mediating role of psychological resilience. The indirect effect accounted for approximately 13.85% of the total effect; thus, Hypothesis 2 was supported.

### 3.4 Gender as a moderator

**Figure 2:**
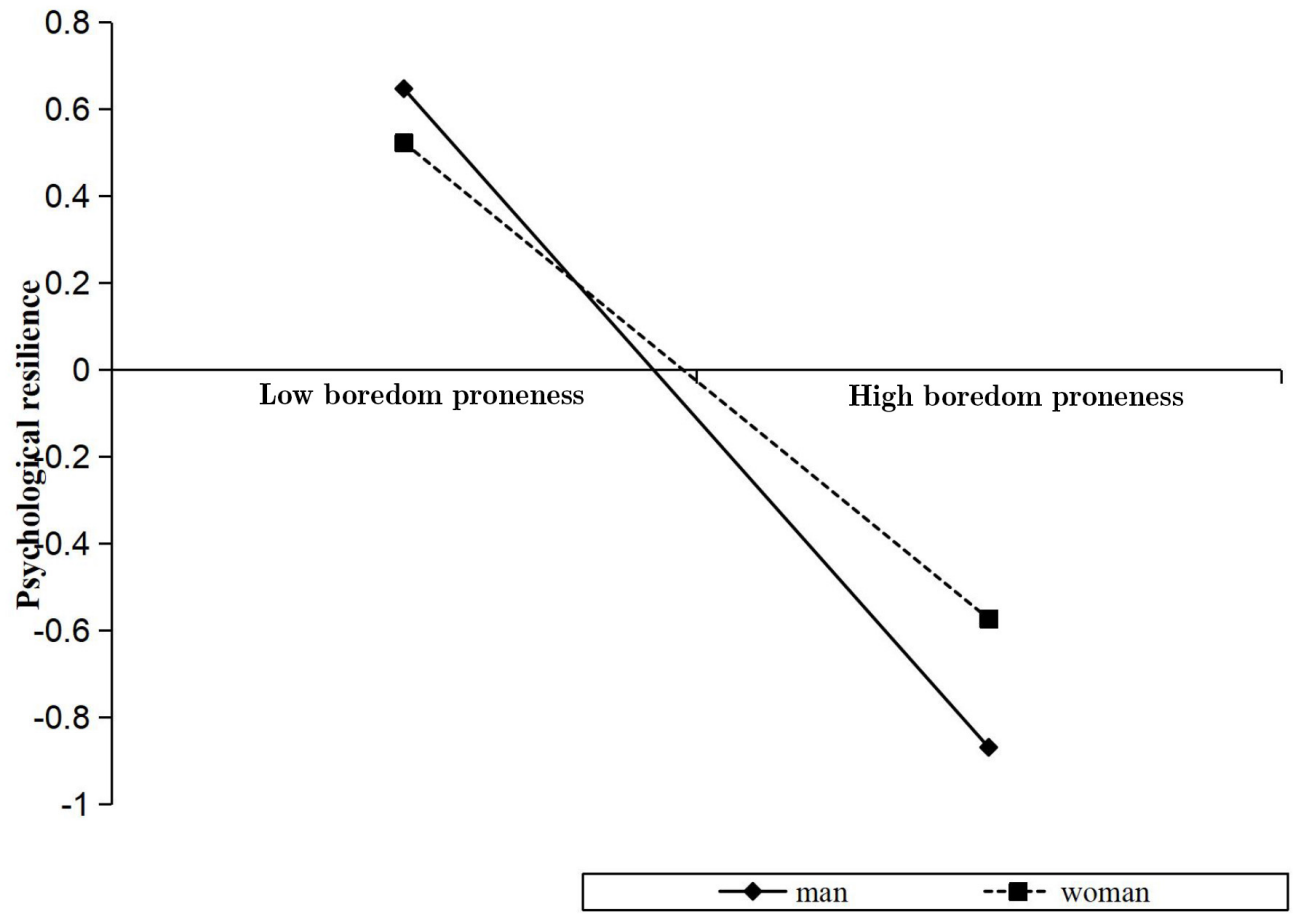
Gender moderates the relationship between boredom proneness and psychological resilience.

We used Model 58 of the PROCESS macro (Hayes, 2013) to examine whether gender moderated the indirect association between boredom proneness and short-video addiction via psychological resilience. Age and year of study were included as covariates. As shown in Table 3, after controlling for these covariates, boredom proneness was significantly and negatively associated with psychological resilience (β = −0.651, SE = 0.060, 95% CI [−0.769, −0.533], *p* < 0.001). The interaction between boredom proneness and gender was also significant (β = 0.103, SE = 0.035, 95% CI [0.034, 0.173], *p* = 0.001), indicating that gender moderated the association between boredom proneness and psychological resilience. As illustrated in Figure 2, simple slope analyses showed that boredom proneness was negatively associated with psychological resilience among both male and female students; however, this association was stronger among males (βsimple = −0.548, *t* = −19.375, *p* < 0.001) than among females (βsimple = −0.444, *t* = −22.175, *p* < 0.001).

**Figure 3:**
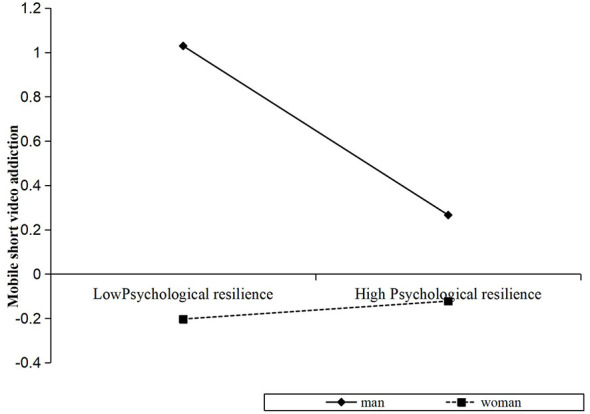
Gender moderates the relationship between psychological resilience and Short-Video addiction.

As shown in Table 3, psychological resilience was significantly and negatively associated with short-video addiction after controlling for age and year of study (β = −0.339, SE = 0.069, 95% CI [−0.476, −0.203], *p* < 0.001). The interaction between psychological resilience and gender was significant (β = 0.171, SE = 0.039, 95% CI [0.094, 0.249], *p* < 0.001), suggesting that gender also moderated the association between psychological resilience and short-video addiction. Simple slope analyses indicated Figure 3 that psychological resilience was significantly and negatively associated with short-video addiction among male students (βsimple = −0.171, *t* = −4.786, *p* < 0.001), whereas this association was not significant among female students (βsimple = 0.005, *t* = 0.173, *p* > 0.05). These findings suggest that the negative association between psychological resilience and short-video addiction was evident only among male students. In addition, the direct association between boredom proneness and short-video addiction remained significant after psychological resilience and the interaction terms were included in the model (β = 0.138, SE = 0.022, 95% CI [0.096, 0.180], *p* < 0.001).

Finally, the bias-corrected bootstrap results based on 5,000 resamples showed that the index of moderated mediation was significant (index = −0.094, Boot SE = 0.026, 95% CI [−0.146, −0.045]). The conditional indirect association between boredom proneness and short-video addiction through psychological resilience was significant among male students (effect = 0.092, Boot SE = 0.024, 95% CI [0.047, 0.141]), but not among female students (effect = −0.002, Boot SE = 0.014, 95% CI [−0.027, 0.026]). Taken together, these results support the proposed moderated mediation model.

## 4 Discussion

This study, based on the analytical framework of “individual susceptibility characteristics–protective psychological resources–individual differences,” examined the relationship between boredom proneness and short-video addiction among vocational college students (Brand et al., 2019; Hobfoll et al., 2018), and tested the mediating role of psychological resilience and the moderating effect of gender. Recent evidence on problematic TikTok and short-video use further supports the relevance of examining short-video addiction through emotional-cognitive and protective-resource mechanisms (Yao et al., 2023; Tian and Wang, 2026). The results showed that vocational college students with higher levels of boredom proneness reported greater short-video addiction. Psychological resilience partially accounted for this association: higher boredom proneness was related to lower psychological resilience, which in turn was associated with greater short-video addiction. The moderated mediation analysis further indicated that this indirect association differed by gender, with the mediating role of psychological resilience primarily evident among male students. Overall, these findings identify psychological resilience as a potential explanatory factor and gender as an important boundary condition in the link between boredom proneness and short-video addiction. Extending previous research on boredom proneness and problematic digital media use, this study situates this association within a resource-based framework of “psychological resource depletion–reduced adaptive regulatory capacity–increased risk of addictive media use” (Camerini et al., 2023; Hobfoll et al., 2018; Tagliaferri et al., 2025). This perspective helps explain how reduced psychological resources may be related to short-video addiction among vocational college students. Given the cross-sectional design, however, these findings should be interpreted as associative rather than causal.

### 4.1 Boredom proneness positively predicts short-video Addiction

This study found that boredom proneness was positively associated with short-video addiction, supporting Hypothesis H1. This finding suggests that boredom proneness may be an important psychological susceptibility factor for short-video addiction among vocational college students. It is also consistent with previous research on problematic digital media use, which has shown that individuals with higher boredom proneness are more likely to report problematic use of smartphones, social media, and short-video platforms (Chou et al., 2018; Elhai et al., 2018; Donati et al., 2022; Lv and Wang, 2023; Malik et al., 2024; Wan et al., 2025; Yang et al., 2026; Zhou et al., 2025). Longitudinal evidence further indicates that boredom proneness can positively predict the development of addictive media use (Zhang L. et al., 2023). Taken together, these findings suggest that boredom proneness is not only a concurrent psychological correlate of short-video addiction, but may also represent a meaningful risk-related factor for problematic media use. This interpretation is further supported by research on problematic TikTok use, which highlights the role of boredom-related and emotion-regulation processes in problematic short-video engagement (Yao et al., 2023).

The results of this study are consistent with the I-PACE model, which suggests that addictive application use develops through the interaction of individual susceptibility factors, affective-cognitive responses, and executive control functions, and may be maintained through reinforcement learning processes. Boredom proneness can be viewed as a relatively stable psychological susceptibility factor. Individuals with higher boredom proneness are more likely to experience low stimulation, reduced sense of meaning, insufficient attentional engagement, and lack of purpose, which may increase their motivation to seek stimulation, emotional relief, and immediate gratification. This association is particularly relevant for vocational college students, who are in a critical transitional stage from school learning to career development and must manage professional skills training, internships, employment preparation, and career planning. When learning engagement is insufficient, course meaning is weak, or campus life is relatively monotonous, short videos may become a convenient way to relieve boredom because they provide instant feedback, rapid content switching, and continuous stimulation. Recent evidence further suggests that short-form video addiction is associated with academic procrastination through attentional-control mechanisms (Xie et al., 2023).

Furthermore, compensatory media use theory suggests that individuals may use media to alleviate negative experiences and compensate for unmet psychological needs (Kardefelt-Winther, 2014). Because short-video platforms offer continuous novelty, instant feedback, and emotional relief at relatively low cost, individuals with high boredom proneness may be more likely to use them as a habitual strategy for emotion regulation or escape. Through repeated reward reinforcement, this pattern may be associated with an increased risk of addictive use. In this sense, short-video addiction is not merely a matter of excessive usage time; rather, it may reflect individuals' reliance on immediate rewards when experiencing boredom, a low sense of meaning, and limited self-regulation (Brand et al., 2019; Kardefelt-Winther, 2014; Tagliaferri et al., 2025). Thus, this study highlights the importance of understanding short-video addiction from the perspective of psychological susceptibility factors.

### 4.2 The mediating effect of psychological resilience

This study found that boredom proneness was significantly and negatively associated with psychological resilience, while psychological resilience was significantly and negatively associated with short-video addiction among vocational college students. In other words, psychological resilience served as a statistical mediator in the relationship between boredom proneness and short-video addiction, supporting Hypothesis H2. These results suggest that boredom proneness is not only directly related to short-video addiction but also indirectly associated with it through reduced psychological resilience. A possible psychological pathway is that individuals with higher boredom proneness may possess fewer psychological adaptation resources, weaker emotion-regulation and stress-coping abilities, and a greater tendency to seek immediate relief and compensatory satisfaction through short videos. This explanation is consistent with recent findings showing that short-form video app addiction is associated with academic anxiety and lower academic engagement among college students (Li et al., 2024).

First, this study found that boredom proneness was significantly and negatively associated with psychological resilience among vocational college students, supporting Hypothesis H2a. This finding is consistent with prior evidence linking boredom proneness to poorer psychological adjustment and lower resilience-related functioning (Bambrah et al., 2022; Akyil, 2025). Individuals with higher boredom proneness often have greater difficulty sustaining attention, maintaining goal persistence, and adapting to monotonous or low-meaning situations (Lee and Zelman, 2019; Bieleke et al., 2022). From a resource perspective, boredom proneness may be regarded as a dispositional risk factor associated with repeated low-stimulation and low-purpose experiences, whereas psychological resilience reflects a personal resource that supports recovery and adaptation in the face of stress or adversity. Conservation of Resources theory is relevant here because it explains how threatened or depleted psychological resources may be associated with poorer adaptive functioning over time, rather than because it directly equates boredom with resilience (Hobfoll, 2015; Hobfoll et al., 2018). For vocational college students, repeated experiences of boredom may be related to weaker learning goals, lower career expectations, and reduced self-regulatory engagement, thereby limiting their capacity to maintain positive adaptation and recover from stress.

Second, psychological resilience was significantly and negatively associated with short-video addiction among vocational college students, supporting Hypothesis H2b. This finding suggests that psychological resilience may serve as an important protective resource linked to lower levels of short-video addiction. Consistent with previous studies, higher psychological resilience has been associated with lower risks of problematic social network use and problematic internet use, and may partly buffer the association between stress and addictive media use (Hou et al., 2017; Hao et al., 2023). Meta-analytic evidence further indicates that higher psychological resilience is associated with a reduced likelihood of uncontrolled digital media use (Hidalgo-Fuentes et al., 2023). According to Conservation of Resources theory, individuals' abilities to cope with stress, regulate emotions, and maintain behavioral control depend partly on the availability of psychological resources. As a key protective resource, psychological resilience may help vocational college students maintain emotional stability, cognitive flexibility, and goal orientation in stressful or boring situations, thereby reducing their tendency to compensate for or escape from these experiences through short videos (Hobfoll et al., 2018; Hou et al., 2017; Wu et al., 2024). Recent short-video addiction research also suggests that psychological resilience can function as a protective pathway in the association between social comparison and short-video addiction (Tian and Wang, 2026). Conversely, students with lower psychological resilience may be more likely to rely on the immediate rewards provided by short videos to relieve negative experiences, and repeated reinforcement of this pattern may be associated with a higher risk of addictive use.

Therefore, the mediating role of psychological resilience suggests that the association between boredom proneness and short-video addiction is not only direct but also indirect through psychological resilience. Specifically, boredom proneness may be linked to fewer stress-coping and emotion-regulation resources, making students more likely to rely on the immediate stimulation, emotional relief, and compensatory satisfaction provided by short videos; this pattern may, in turn, be associated with higher levels of addictive use. This finding extends previous research on boredom proneness and short-video addiction, most of which has emphasized the direct predictive role of boredom proneness in problematic media use. The present study further indicates that psychological resilience may be an important protective resource connecting boredom proneness with short-video addiction. Thus, short-video addiction can be understood from the perspective of the combined association between risk factors and protective resources: students with higher boredom proneness and lower psychological resilience may be more likely to seek immediate satisfaction and emotional relief through short videos, thereby increasing their risk of addictive use.

### 4.3 The moderating role of gender

The results showed that gender moderated the association between boredom proneness and psychological resilience among vocational college students. Specifically, the negative association between boredom proneness and psychological resilience was stronger among male students than among female students, supporting Hypothesis H3a. This finding indicates that this association is not uniform across gender but shows gender-specific differences. It further suggests that psychological resilience, and its relationship with boredom proneness, may be influenced not only by individual psychological traits but also by gender-role expectations, emotional expression patterns, and the use of social support.

This result can be interpreted through gender-role socialization theory, which suggests that men and women may develop different patterns of emotional expression, stress coping, and support seeking because of different social expectations and role norms (Rosario et al., 1988; Matud, 2004; Tamres et al., 2002). Compared with female students, male students may be more likely to internalize boredom, emptiness, or low-arousal negative states, and may be less likely to regulate these experiences through open emotional expression or interpersonal support. In this context, boredom proneness may be more strongly associated with lower adaptive psychological resources among male students. This pattern may be further strengthened in the vocational education context, where students simultaneously face skill acquisition, career planning, internship preparation, employment pressure, and identity development (Xie et al., 2023; Li et al., 2024). For male students, role expectations related to achievement, independence, and self-confirmation may make boredom-related frustration more closely associated with reduced perceived control and lower psychological resilience. Overall, these findings suggest that the association between boredom proneness and psychological resilience should be understood in relation to both individual traits and gendered coping patterns within the vocational education context.

However, this interpretation should be treated with caution. Previous research suggests that gender differences in emotional regulation, coping, and problematic media use are not always strong or consistent, but may depend on social, cultural, and contextual factors (Hyde, 2005; Zell et al., 2015). Therefore, the stronger association observed among male students should not be interpreted as evidence of inherent male vulnerability. Rather, it may reflect the specific vocational education context, sample characteristics, and the meaning of boredom and resilience in short-video use.

The results also showed that gender moderated the association between psychological resilience and short-video addiction. Specifically, the negative association between psychological resilience and short-video addiction was significant among male students but not among female students, supporting Hypothesis H3b. This finding should not be interpreted as indicating that gender itself is either a risk factor or a protective factor. Rather, gender appears to function as a boundary condition that changes the extent to which resilience is associated with problematic short-video use. Within the Protective Factor Model, psychological resilience can be regarded as an internal protective resource associated with better regulation of negative affect, stronger cognitive and behavioral control, and a lower likelihood of compensatory or avoidance-based short-video use (Hou et al., 2017; Hao et al., 2022; Hidalgo-Fuentes et al., 2023). The stronger negative association observed among male students may reflect their greater reliance on internal regulatory resources when external emotional expression or support seeking is constrained. In contrast, female students' short-video use may be more closely related to interpersonal connection, emotional expression, peer interaction, social comparison, or other relational motives, which may weaken the independent association between resilience and short-video addiction in this pathway. This explanation is supported by recent evidence showing that gender differences may emerge in pathways linking upward social comparison, negative emotions, psychological resilience, and short-video addiction (Tian and Wang, 2026). Nevertheless, the non-significant association among female students should be interpreted with caution. It does not indicate that psychological resilience is unrelated to female students' short-video addiction. Instead, this association may be weaker, more indirect, or more dependent on interpersonal and contextual factors. Thus, the present findings should be understood as sample-specific rather than as definitive evidence of stable gender differences.

Compared with male vocational college students, female students' short-video use may be more closely associated with interpersonal connection, emotional expression, social interaction, and peer support. Therefore, even among female students with high psychological resilience, the negative association between psychological resilience and short-video addiction may be less pronounced when short-video use is embedded in social relationships or emotional expression patterns. This may help explain why this association did not reach statistical significance in the present study. However, this result does not imply that psychological resilience is unimportant for female students. Rather, it suggests that the association between psychological resilience and short-video addiction among female students may not operate primarily through an independent resilience-related pathway, but may also involve emotional expression, interpersonal interaction, social comparison, peer relationships, and media-use motivation. Future research should further examine social-comparison and resilience-related pathways in gender-specific mechanisms of short-video addiction (Tian and Wang, 2026). Future longitudinal and cross-cultural studies are needed to determine whether these gender-related patterns can be replicated across different student groups, educational contexts, and short-video platforms.

## 5 Research implications

This research offers several theoretical and practical implications. Theoretically, this study integrates boredom proneness, psychological resilience, and short-video addiction into a single analytical framework, revealing a psychological pathway through which boredom proneness is associated with short-video addiction. The results indicate that boredom proneness is not only positively related to short-video addiction but may also be indirectly linked to problematic short-video use through lower psychological resilience. This finding deepens the understanding of the psychological mechanisms underlying short-video addiction among vocational college students and extends the application of the I-PACE model and Conservation of Resources theory to short-video addiction research.

Practically, this study highlights the protective value of psychological resilience. Interventions for short-video addiction among vocational college students should not rely solely on external supervision or usage restrictions, but should also focus on strengthening students' psychological resources. Higher vocational colleges can improve students' resilience and self-regulation in situations characterized by boredom, stress, and low motivation through mental health education, emotion-regulation training, stress-management counseling, and the cultivation of positive coping skills. For example, resilience-based intervention programs may include cognitive reappraisal training, mindfulness exercises, goal-setting guidance, problem-solving training, and peer-support activities, which may help students cope with boredom and negative emotions without relying excessively on short videos.

In addition, campus-based boredom management programs may be useful for reducing problematic short-video use among vocational college students. Such programs could help students identify boredom triggers, improve attention regulation, develop meaningful leisure alternatives, and strengthen their sense of learning purpose. At the same time, schools can reduce the likelihood that students remain in states of low stimulation and low engagement by optimizing curriculum design, enriching practical teaching, enhancing the perceived significance of learning tasks, and providing career development support. For vocational college students in particular, practice-oriented courses, career-planning workshops, skill competitions, internship preparation, and structured extracurricular activities may provide meaningful engagement opportunities and reduce boredom-related reliance on short videos.

Finally, this study suggests that interventions for short-video addiction should take gender differences into account. For male vocational college students, emphasis should be placed on strengthening training in psychological resilience, emotional expression, and support-seeking abilities to help them establish more positive emotion regulation and stress-coping patterns. For female vocational college students, comprehensive intervention should be carried out by combining interpersonal support, emotion management, social comparison adjustment, and motivation guidance for media use. However, gender-specific intervention should avoid stereotyping students and should instead be based on individual psychological needs, coping styles, and media-use motivations. In conclusion, higher vocational colleges should avoid adopting a one-size-fits-all intervention approach. Instead, they should provide more targeted support based on the psychological characteristics, coping styles, and media usage motivations of students of different genders.

## 6 Limitations and implications

This study has several limitations. Firstly, regarding sample and external validity, the participants were primarily drawn from vocational colleges in Guangxi, China, which may limit the generalizability of the findings to other regions, types of institutions, or student populations with different educational backgrounds. In addition, the sample distribution was not fully balanced across demographic categories, which may also affect the representativeness and external validity of the findings. Future research can expand the sample sources by including students from different provinces, different regions and various types of vocational colleges. Moreover, more rigorous sampling procedures such as stratified sampling or multi-stage sampling can be adopted to enhance the external validity of the research results.

Second, this study has limitations in research design and causal inference. Because a cross-sectional design was adopted, it is not possible to strictly determine the causal relationships or temporal sequence among boredom proneness, psychological resilience, and short-video addiction. Future research could use three-wave or multi-wave longitudinal designs and apply cross-lagged models or latent growth models to examine the dynamic temporal relationships between boredom proneness and short-video addiction. For example, future studies could test whether boredom proneness predicts subsequent increases in short-video addiction, or whether short-video addiction, in turn, intensifies individuals' boredom. This potential bidirectional relationship deserves attention, because problematic short-video use may reduce learning engagement, disrupt time management, and weaken participation in real-life activities, thereby further increasing boredom and creating a vicious cycle. In addition, experimental studies could induce boredom-related situations to examine how state boredom affects impulsive short-video use and immediate usage behavior, thereby strengthening causal inference.

Finally, there are limitations in the measurement methods and psychological mechanisms. All key variables in this study, including boredom proneness, psychological resilience, gender, and short-video addiction, were measured using self-report questionnaires. This measurement approach may introduce common method bias and social desirability bias, as participants may provide responses that are consistent with their self-perceptions or socially acceptable expectations. Although the test for common method bias indicated that the problem was not significant, the possibility of common method variance cannot be completely ruled out. For instance, students might underreport their problematic short-video use or respond based on their overall tendency of self-evaluation. Future research can integrate objective screen usage data, platform usage logs, reports from peers or teachers, as well as behavioral measurements to enhance ecological validity and the robustness of results. Furthermore, this study only examined the mediating role of psychological resilience and the moderating effect of gender. There may be other psychological mechanisms and boundary conditions between boredom proneness and short-video addiction. In the future, variables such as self-control, impulsivity, social comparison, fear of missing out, and difficulty in emotional regulation can be included to examine their mediating or moderating effects, in order to more comprehensively reveal the risk mechanism of short-video addiction.

## Data Availability

The raw data supporting the conclusions of this article will be made available by the authors, without undue reservation.
